# On the Impact of the Data Acquisition Protocol on ECG Biometric Identification

**DOI:** 10.3390/s21144645

**Published:** 2021-07-07

**Authors:** Mariana S. Ramos, João M. Carvalho, Armando J. Pinho, Susana Brás

**Affiliations:** 1 Department of Physics, University of Aveiro, 3810-193 Aveiro, Portugal; marianasr@ua.pt; 2 Department of Electronics Telecommunications and Informatics, University of Aveiro, 3810-193 Aveiro, Portugal; joao.carvalho@ua.pt (J.M.C.); ap@ua.pt (A.J.P.); 3 IEETA-Institute of Electronics and Informatics Engineering of Aveiro, University of Aveiro, 3810-193 Aveiro, Portugal

**Keywords:** biometric identification, biometric sensing, ECG, data acquisition

## Abstract

Electrocardiographic (ECG) signals have been used for clinical purposes for a long time. Notwithstanding, they may also be used as the input for a biometric identification system. Several studies, as well as some prototypes, are already based on this principle. One of the methods already used for biometric identification relies on a measure of similarity based on the Kolmogorov Complexity, called the Normalized Relative Compression (NRC)—this approach evaluates the similarity between two ECG segments without the need to delineate the signal wave. This methodology is the basis of the present work. We have collected a dataset of ECG signals from twenty participants on two different sessions, making use of three different kits simultaneously—one of them using dry electrodes, placed on their fingers; the other two using wet sensors placed on their wrists and chests. The aim of this work was to study the influence of the ECG protocol collection, regarding the biometric identification system’s performance. Several variables in the data acquisition are not controllable, so some of them will be inspected to understand their influence in the system. Movement, data collection point, time interval between train and test datasets and ECG segment duration are examples of variables that may affect the system, and they are studied in this paper. Through this study, it was concluded that this biometric identification system needs at least 10 s of data to guarantee that the system learns the essential information. It was also observed that “off-the-person” data acquisition led to a better performance over time, when compared to “on-the-person” places.

## 1. Introduction

Personal identification is crucial nowadays. However, how to univocally identify someone is still a concern [1,2]. There are several strategies that may involve passwords or systems based on biometric data [2]. Usual examples are the fingertips, iris and face. There is no infallible system; nevertheless, it is intended to find one that may be more secure. Systems that do not guarantee liveliness, nor need contact with the subject, are more susceptible to being falsified [2]. This area has been extensively investigated, since this type of identification offers high security because the measurability is guaranteed, in most cases, and the evasion and manipulation of the data are more difficult, given the complexity of an individual manipulating their own physiological signals [1,2]. So, there is a considerable interest in using physiological signals for biometric identification [1]. The electrocardiogram (ECG) signal is an example of such systems [1,2,3,4]. The ECG possesses an inter-variability, that is the source of richness that prints the univocal key on ECG, making it an interesting signal for biometric applications.

The recognition of each individual is valid when the technique used presents some particularities, such as universality, permanence, singularity, reproducibility, performance and acceptability of the data. Universality guarantees that the characteristic in question is common to the majority of the population. ECG recognition would solve some problems associated with the acquisition of biomedical signals, since everyone has a heartbeat and its collection is accessible. For this reason, this method is beneficial in relation, for example, to identification through the fingerprint, since there are people without upper limbs who do not have this facility [5]. Permanence ensures that the characteristic collected is constant, that is, that the properties will not change over time. The uniqueness allows that each ECG segment is relative to only one individual, and therefore that each person has a unique ECG. Reproducibility guarantees the ease of data acquisition using similar protocols and conditions. Another factor that must be taken into account is the performance of the method. It might be measured using different metrics, with the aim of assessing how close the method’s predictions are to being correct. Finally, acceptability certifies the viability of the biometric identification system, where one of the important factors is the computational cost [1,5].

ECG is affected by external variability and interferences which affect the signal and its quality. Therefore, to grasp the ECG univocal key, the methods should be robust to fluctuations. The system should also correctly deal with variability and different sources of noise—muscle, movement, electrodes placement or pathologies [1,2]. The application of parameter free methods, such as compression algorithms, have been proved efficient in classification, since there is no pre-assumption about the premises, allowing true exploratory data mining [6].

In 2018, we introduced a method for ECG biometric identification, based on relative compression [4]. Although satisfactory results were obtained, we felt the need to test the method under as real conditions as possible, taking into account some of the variables that we were unable to control. The idea behind this method is to estimate the Kolmogorov complexity of an ECG signal using compression algorithms. However, since the ECG is not symbolic, we also perform quantization according to a scheme introduced in [7], which uses the first derivative of the original signal as the input. The method presented a good performance; nevertheless, the time needed for data acquisition was too high to be considered with real scenarios. Therefore, it is essential to validate how this system performs when the data acquisition time is different and more realistic, such as using one to five second signal segments. Methods can also currently have their performances conditioned by external non controlled conditions, such as physiological (caused by coffee, stress and cardiac irregularities, namely, premature ventricular contraction), behavioral (such as muscle and environmental movement) and contraction, such as the placement of electrodes and the temperature.

So, in this work, the system performance is tested when data are acquired considering different premises: dry electrodes and wet electrodes, and data acquired at different body positions and movements. The main contribution of this paper is to evaluate the impact of the input’s initial conditions variability on biometric system performance. Moreover, it is expected that, as the ultimate goal, the achieved results may contribute to the design of feasible data collection protocols for ECG biometric systems.

## 2. Dataset

The effectiveness of recognizing an individual through his ECG depends, amongst other things, on the conditions to which he is exposed during the acquisition process. Therefore, it is extremely important to assess the impact that certain changes have on the biometric identification results. This dataset was built with the purpose of analyzing the influence of changes—such as movement, day of data collection and location of the body on which the electrodes are placed, as well as the time of signal acquisition—on the performance of biometric identification.

This study was approved by the ethics council of the University of Aveiro. Given that the application is biometric identification, two mandatory prerequisites were to perform ECG collections from different individuals and to have more than one session per participant. Data were collected from twenty healthy participants (nine females and eleven males), all between the ages of 20 and 23 years (20.85 ± 0.91 years). All participants were healthy, without any relevant pathology. Two of the participants were smokers. The participants, recruited at the University of Aveiro, gave their written consent, after the purpose of the data collection was explained to them, as well as how the data would be used. It was also mentioned to them that the data were confidential and that they could withdraw from the experiment at any time.

Electrodes are responsible for measuring the electrical activity of the heart, recorded in potential difference, across the surface of the skin [8]. These consist of a metallic surface and an electrolyte in contact with the skin, thus creating two interfaces: the metal–electrolyte and the skin–electrolyte. Ideally, it is intended that the electrodes have excellent contact with the skin so that the impedance is minimal, as well as the sensitivity to the impact of the action. However, ECG is a vital signal, which can be affected by factors such as the skin and the type of electrodes used [9]. For this reason, two types of electrodes were used: dry ones, produced by BITalino and gel ones, manufactured by Ambu^®^, both of the Ag/AgCl type.

The biomedical device used to collect the ECG was the Vital Jacket^®^, a scientifically validated biomedical device developed by a technological company called Biodevices SA, in partnership with researchers from IEETA, a research unit at the University of Aveiro. The data were collected at 500 Hz, using an Android application called DroidJacket, which allowed the collection of data from different devices and their redirection to a storage location [10].

The Ag/AgCl type gel electrodes that were used have a gel that improves conductivity and reduces the impedance of the skin–electrode interface. However, dry electrodes were placed on the fingers with the aim of making this study the closest to reality. In daily life, the ideal would be to use dry electrodes in an easily accessible area, without the need to place gel electrodes, since the latter have a more complex placement process and they are also subject to the drying of the gel, leading to a drastic decrease in the quality of the signal, especially when dealing with long acquisition processes [11].

**Figure 1:**
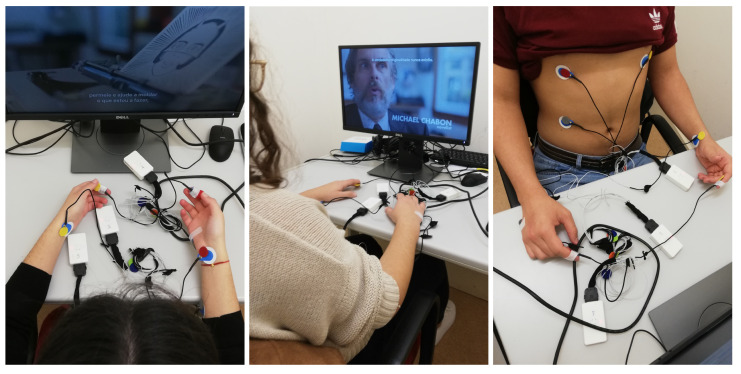
Example of electrode placement on both hands, wrists and chest.

The ECG signals were acquired through a rigorous placement of each electrode in different regions of the body: fingers from both hands, wrists and chest (shown in Figure 1). In each region of the body from which the ECG signal was collected, three electrodes were placed. Their placement was uniform, since for each of the body locations and for each of the participants, the exact same placement protocol was followed. These electrodes were attached to the end of the wires with different colors, facilitating the process. The red and yellow color electrodes were always placed on a high blood flow region, on the right and left sides, respectively. The third electrode, called neutral, was always placed on the right side of the body—in a place with less blood flow. In this way, it was possible to collect signals from different collection places simultaneously.

Two data acquisition sessions took place. In both collections, each participant underwent an initial survey with the main objective of evaluating some factors that, according to the literature, can alter the biometric identification “signature” [2]: their levels of anxiety and stress, their last intake of coffee and also if they had any disease. Afterwards, participants watched a documentary, which showed a neutral feeling, thus not introducing any specific emotion. At the end of each collection, a satisfaction survey was completed.

In the first data collection session, each participant was asked to remain at rest for ten minutes. After this period of time, they were asked to perform movements with their hands, feet and torso, for two minutes each, with a pause of one minute between them. In the second session, participants were asked to stay for five minutes at rest while signals were being collected.

The two collections carried out had a time spacing of two weeks. This period was rigorously chosen to assess the impact of the temporal distance of data collection for biometric identification.

## 3. Methods

In order to perform biometric identification of an individual, several steps are required. In this section, we explain which methods were used for the main steps of the workflow. We start explaining how the data was pre-processed. Afterwards, we introduce the finite-context models, the basis for the compressors used. We then finish the section with the definition of the Normalized Relative Compression and how it was used to compute relative similarities, as well as some definitions that are necessary to understand why they make sense from a theoretical perspective.

### 3.1. Pre-Processing Steps

**Figure 2:**
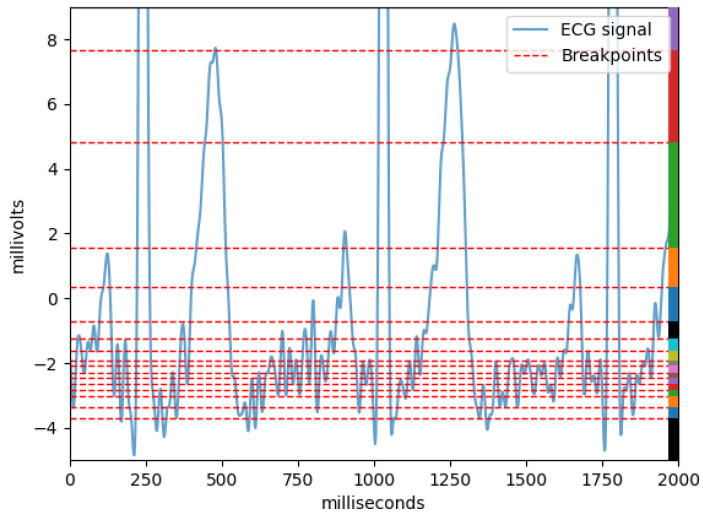
Quantization scheme example on two seconds of ECG signal. The ECG signal is represented by a blue line and the red dashes represent the breakpoints learned by the Lloyd–Max quantizer for this two second interval. The colors on the right are merely illustrative to help visualise the different intervals. Each interval represents a different symbol used after the quantization is done, i.e., when each sample on the signal is replaced by the corresponding symbol from the quantization interval it belongs to.

When working with non-symbolic data, it is usual to perform a preprocessing step of sampling and/or quantization before any data compression is done. In this work, we decided to use the original sampling rate of the data (500 Hz). Since the ECG signal suffers from baseline wander, we have also chosen to operate on its consecutive differences, instead of on the original signal, and use a Lloyd–Max quantization scheme on top of the signal—both on the training and testing data separately, avoiding data leakage from training to test [12]. More details on this quantization method can be found in [7], but the basic idea is that the quantizer learns breakpoints so that each symbol on the output will have approximately the same number of samples, meaning that regions with higher probability will be lower in interval range and vice versa. The quantizer then replaces each of the original samples with the symbol corresponding to the interval it belongs to, meaning that a sequence of floating numbers becomes a sequence of symbols (in our implementation (the source code for the quantizer was implemented from scratch using Python 3.7 and we made it openly available on https://github.com/joaomrcarvalho/diffquantizer), capital letters). An example of such a quantization can be seen in Figure 2.

### 3.2. Compressing Using Finite-Context Models

Finite context models (FCMs) have been useful in very different pattern recognition tasks, namely, using DNA sequences [13], text [14], images [15] and ECG signals [4,7,16,17,18,19]. Recent work has shown that they have the ability to measure similarity/dissimilarity, relying on the data algorithmic entropy [20].

An FCM complies to the Markov property, that is, it estimates the probability of the next symbol of the information source using the k>0 immediate past symbols (order-*k* context) to select the probability distribution. Therefore, assuming that the *k* past outcomes are given by $x_{n-k+1}^{n}=x_{n-k+1}\cdots x_{n}$, the probability estimates, $P(x_{n+1}|x_{n-k+1}^{n})$, are calculated using symbol counts that are accumulated while the information source is processed, with

(1) $$P\left(s|x_{n-k+1}^{n}\right)=\frac{v(s|x_{n-k+1}^{n})+\alpha }{\sum_{a\in A}v\left(a|x_{n-k+1}^{n}\right)+\alpha \left|A\right|},$$

where $A=\{s_{1},s_{2},\cdots s_{\left|A\right|}\}$ is the alphabet that describes the objects of interest, $v(s|x_{n-k+1}^{n})$ represents the number of times that, in the past, symbol s∈A was found having $x_{n-k+1}^{n}$ as the conditioning context. The parameter α allows balancing between a maximum likelihood estimator and a uniform distribution—it can also be seen as a smoothing factor over the possible outcomes.

After processing the first *n* symbols of *x*, the total number of bits generated by an order-*k* FCM is given by

(2) $$-\sum_{i=1}^{n}\log_{2}P\left(x_{i}|x_{i-k}^{i-1}\right).$$

For compressing the first *k* symbols of a sequence, we do not have enough symbols to represent a context of length *k*. For that reason, we always assume that the sequence is circular [21]. For long sequences, such as a previously quantized ECG signal, this should make a negligible difference in terms of the number of bits required to perform the relative compression. The software used for that purpose is publicly available for download (the source code was implemented using Python 3.6 and is publicly available under the GPL v3 license at https://github.com/joaomrcarvalho/xafcm).

### 3.3. Computing Relative Similarity with the Normalized Relative Compression (NRC)

The Kolmogorov complexity of a binary string of finite length *x*, K(x) is the length of a shortest binary program $x^{*}$ that computes *x* in a universal Turing machine and halts. Therefore, $K\left(x\right)=|x^{*}|$, the length of $x^{*}$, represents the minimum number of bits from which *x* can be computationally retrieved [22].

In 1998, Bennett et al. [23] proposed the Information Distance (ID) as well as the Normalized Information Distance (NID), its normalized version. These metrics are defined in terms of the Kolmogorov complexity of the strings involved, as well as the complexity of one when the other is provided. The limitation of this approach is that the Kolmogorov complexity of a string is generally not computable. An approximation (upper bound) for it can be used by means of a compressor, which explores the possible redundancies of a string. Let C(x) be the number of bits used by a compressor to represent a binary string *x*. In this work, we used a measure called the Normalized Relative Compression (NRC), based on the notion of relative compression [14], denoted by C(x||y), which represents the compression of *x* relative to *y*.

This measure complies with these rules:

- C(x||y)≈0 iff string *x* can be built efficiently from *y*;
- C(x||y)≈|x| iff K(x|y)≈K(x).

Based on the previous rules, the NRC of string *x* relative to string *y* is defined as

(3) $$\mathrm{NRC}\left(x||y\right)=\frac{C(x||y)}{\left|x\right|},$$

where |x| denotes the length of the string *x*.

## 4. Results

The aim of this work was to evaluate how different variables from the data acquisition protocol interfered in the biometric identification task. For that purpose, the first step carried out was to choose the parameters for the preprocessing steps, namely, the quantization, as well as for the models used to compute the number of bits needed for the compression. Several tests were carried out for that purpose. For the preprocessing of the data, we tried both a Butterworth low-pass filter of order eight at 40 Hz cutoff frequency and no filtering whatsoever. For the quantization, we decided to use a fixed alphabet of 20 symbols, as in a previous study [4]. Finally, regarding the finite context models used, we performed experiments both using cooperative models (mixtures of finite context models [24]) and single *k* value models. The single models were more accurate in general, so we opted to use them for the rest of the experiments.

After analyzing the results from these experiments, the parameters were chosen for performing filtering using the Butterworth filter mentioned, to use a single finite context model with order k=20 and α was set to ‘auto’ (see [17] for more details). These parameters were used in all the experiments shown in this section.

The biometric identification of an individual using physiological signals can be affected by external variabilities such as the movement an individual makes during the data collection, the session (or sessions) where the data collection took place, amongst other factors, such as the noise present on the signal [25] and the duration of acquisition [26]. In order to study the impact of different changes during collection, it was decided to carry out different tests for which external variability was introduced.

### 4.1. Measuring the Influence of Movement

**Figure 3:**
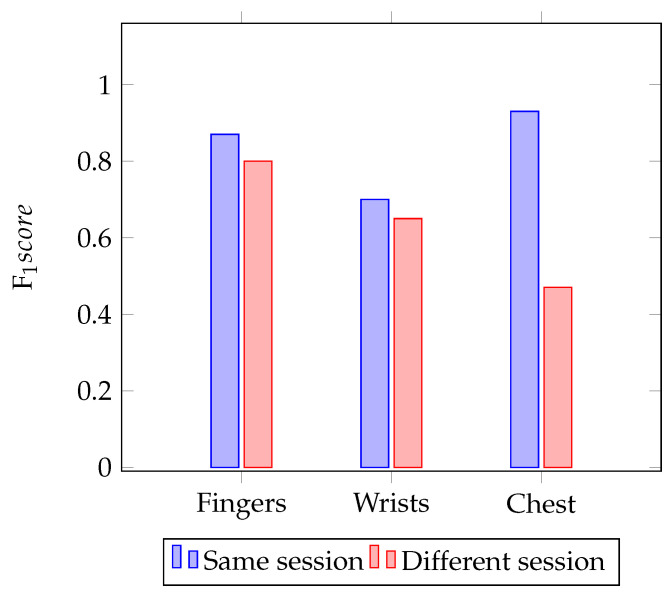
Histogram of the accuracy values, for the same and different sessions in every single kit.

As a first approach, the impact of moving hands, feet and chest was assessed. For this study, we used two minutes of data for testing different movements and we trained the model with participants at rest for eight minutes, during the same session. According to the results presented in Table 1, the movement of the wrists causes the biggest error in biometric identification, followed by the movement of the fingers. On the other hand, the movement of the chest has almost no impact on the performance of the method. These results can be justified by the distance from the electrodes to the heart. It is important to mention that both the training and test data from Table 1 contain segments from the same session. This was done with the intent of controlling some variables, since the objective was to study the impact of the variability caused exclusively by the source of movement. If that did not happen, the expected error would be higher. The reason for this can be easily seen in the results shown in Figure 3.

### 4.2. Comparing the Influence of Using Different ECG Acquisition Placement

The majority of publicly available ECG databases contain ECG collected on the chest. Notwithstanding, when a biometric identification system is planned for real practice, data collected on the chest may not be the more appropriate or comfortable setup. The underlined question is: how does the system perform in these conditions? Therefore, it is important to quantify the impact of the use of ECG collected on the chest in the training of the biometric system, when the system is planned to be used with off-the-person sensors.

To obtain comparable results, data in this experiment were collected simultaneously at three points: chest, wrists and fingers. The model was designed with eight minutes of resting chest ECG data, and tested with two minutes of resting condition data taken from the wrists and fingers. An error of 95% was obtained in both, showing that it is not feasible to compare ECG segments from different acquisition points. The reliability of the method decreases, indicating a bias of the model to the body region where data were collected.

### 4.3. Measuring the Influence of Same/Different-Sessions

Temporal separation between biometric evaluations may influence the system’s performance. This influence was studied by the evaluation of ECG segments from the same/different sessions. The model was built with eight minutes of rest period from the first session, and was evaluated on two minutes of rest condition from both sessions, separately.

Figure 3 shows a histogram with the $F_{1}score$ values obtained when testing ECG segments with the participant at rest, both from the same session (and from different sessions of the training) for each of the collection kits. Based on the histogram, it can be seen that there is a decrease in performance when the sessions are different, except when the signal is collected on the fingers.

According to both the histogram and Table 2, it is possible to infer that the signal acquired on the fingers shows greater stability in the long term. On the other hand, the point of acquisition that presents the greatest decrease in performance is the chest. This verified that the instability when the data are collected on the chest may be due to the location of the electrodes. Since the placement area is broader, this leads to an increase in placement error, given that the electrodes may not always be placed in the exact same area. As for the fingers, the area is always the same, since it is much more restricted and there is less margin for error. These results show an enormous potential for the use of electrodes “off-the-person”, such as, for example, the electrodes placed on the fingers, to the detriment of the placement “on-the-person”, which makes the entire data acquisition system more intrusive when it comes to biometric identification methods.

The analysis performed indicated that, when the model is trained with the eight minute rest condition from session 1 and evaluated with the two minute rest condition from session 2, there is an increase in error, as shown in Figure 3. This increase in error can be justified, since it is known that the ECG undergoes circadian changes, associated with the person’s condition. Even a small difference in the electrode’s position can alter the morphology of this wave.

### 4.4. Impact of the Acquisition Time

Finally, tests were carried out in order to assess the impact of the duration of the ECG segment under study on the biometric identification’s performance. Initially, segments from the same data collection are evaluated, that is, both the training and the test belong to the first ECG acquisition session. Next, we study the impact that the duration of the test segments has when trying to recognize an individual with an ECG sample from a different data collection session other than the one used to train the model. To study the impact of acquisition time, the previously used two minutes’ resting ECG segments were divided into the desired time intervals, namely, 60, 30, 15, 10, 8, 6, 4 and 2 s. In this case, segments from different sessions were used because they represent a more realistic situation for a biometric identification system.

As we can see in Figure 3, the performance of the method decreases as the temporal interval between sessions increases. Tests with different duration segments were performed, both from the same session as well as from different sessions. Globally, the performance of the model with segments from the same session was better than when segments from different sessions were analyzed.

In a real situation, we want to be able to identify an individual based on his ECG, independently of the training data collection time. Based on these observations, we conclude that it will be more advantageous to study the influence of the duration of the ECG signal acquisition time when testing segments of the second data collection, using only the first session’s data as training. In this database, a segment of eight minutes of rest from the first data collection was used to train the model.

**Figure 4:**
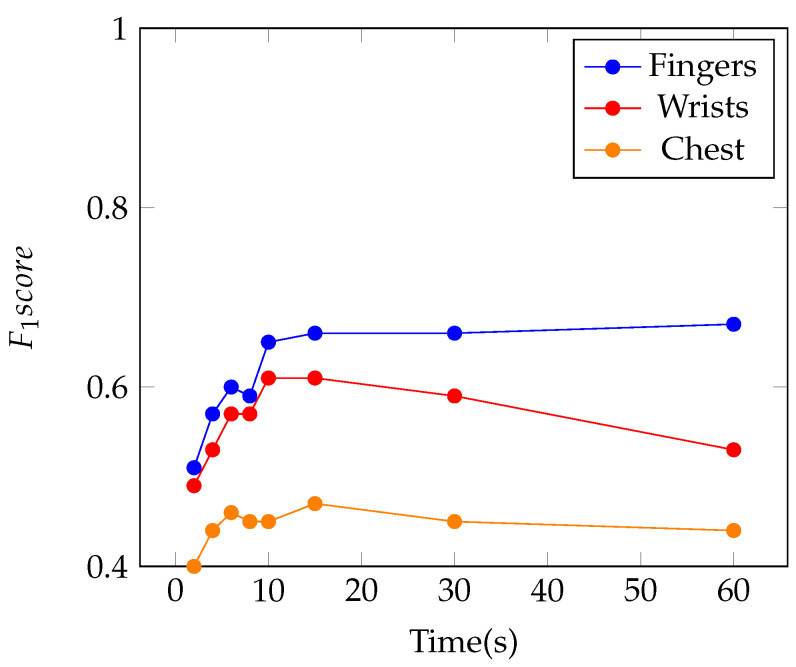
Plot of F1-score as a function of the duration time of the ECG segments from different sessions.

Since the experiment using different sessions portrays a more realistic circumstance, it was decided to increase the number of different acquisition durations to be tested, including shorter segments. Therefore, the impact of the duration was studied, with samples from two seconds up to one minute, as can be seen in Figure 4.

In the literature, it is predicted that the shorter the duration of the ECG segment used, the lower the performance obtained by the system [2]. This behavior was observed, in general, in all kits. However, this conclusion may not always be valid from a certain point onwards, as more data might just introduce redundancy to the system [26].

Nevertheless, it is considered that using approximately ten seconds is the ideal time for a balanced compromise between time and performance, for a system that aims at biometric identification in real time; this was already shown in [26]. In the following graph it can be seen that the decrease in performance observed from six seconds to eight seconds is minimal; 0.01 for the fingers and chest, and this remains the same for the wrists. In the time intervals of eight and ten seconds, increases in the performance of the method of 0.06 and 0.04 are observed, in the fingers and wrists, respectively, remaining constant in the chest. The performance of the system when segments larger than 10 s are used presents a different profile if data were collected on the chest, wrists or fingers. These different behaviors may be due to three combined reasons: the method evaluates relative similarity between two segments and it may be more difficult to evaluate the similarity when the ECG segments are larger, as a small variation along a small portion of the testing sample might affect the final result; the stochastic characteristics of the sample may influence this result, since the statistical representation of the data may vary through time; and the electrodes used on the fingers are dry electrodes, while on the wrists and chest wet electrodes were used.

## 5. Final Remarks

This study aimed to understand the impact of some variables from the data acquisition protocol of an ECG signal in the biometric identification process. This way, we can provide some guidelines when building a dataset for biometric identification.

After studying some of the determining factors that have an impact on the performance of a biometric identification system, we concluded that one of the most important ones is the fact that training and testing data should never be from the same session, as that leads to overoptimistic results, which may not be useful in practice. We have also shown that movements further away from the electrode placement area have less impact on the biometric identification performance than those near it. This is consistent with common sense, but provides scientific validity and should be taken into account when developing an ECG data acquisition protocol—for example, having participants fill surveys while ECG is being collected from their wrists should be avoided. In this study, the impact of the movement and different data collection sessions were accessed independently, since it was intended to quantify the sources of variability controlling other variables. Nevertheless, from our analysis, a combination of changes in the data may represent a larger decrease of the system’s performance than by the evaluation of each change independently.

We also established the need to build and test datasets using the same electrode placement for training and testing a biometric identification system. This might seem obvious. However, some studies for ECG biometric identification use publicly available databases that have been collected for clinical purposes, such as the MIT–BIH Arrhythmia Database or the PTB Diagnostic ECG Database from Physionet [27], amongst others. In those databases, wet gel electrodes were placed on the chest to acquire the signals. It makes sense to use those databases for research and compare results with other state-of-the-art methods, but it is important to understand that the models learned using these datasets might not work as intended with a real application, which should use a less intrusive protocol, such as dry electrodes on the fingers.

Regarding the acquisition time, the results show that around 10 s of signal are enough to test the identity of an individual. After this period of time, the gains in performance are not substantial.

This analysis allowed us to understand and prove that the biometric identification systems should be adaptable to the context, in order to maximize their usability and performance.

## Figures and Tables

**Table 1:** Evaluation of accuracy, error, sensitivity, specificity and $F_{1}score$ for each electrode placement, subject to a certain type of movement, using ECG signals from the same session.

Movement	Electrode Placement	Accuracy	Error	SEN	SPEC	$F_{1}\mathrm{score}$
Hands	Fingers	0.96	0.40	0.60	0.98	0.50
	Wrists	0.94	0.65	0.35	0.97	0.31
	Chest	1.00	0.00	1.00	1.00	1.00
Feet	Fingers	0.99	0.10	0.90	0.99	0.87
	Wrists	0.98	0.20	0.80	0.99	0.75
	Chest	1.00	0.00	1.00	1.00	1.00
Chest	Fingers	0.98	0.25	0.75	0.99	0.69
	Wrists	0.97	0.35	0.65	0.98	0.60
	Chest	1.00	0.05	0.95	1.00	0.93

**Table 2:** Evaluation of accuracy, error, sensitivity, specificity and $F_{1}score$ for each electrode placement, when the method is tested and trained with segments from the same session and different sessions.

Session	Electrode Placement	Accuracy	Error	SEN	SPEC	$F_{1}\mathrm{score}$
Same session	Fingers	0.99	0.10	0.90	0.99	0.87
	Wrists	0.98	0.25	0.75	0.99	0.70
	Chest	1.00	0.05	0.95	1.00	0.93
Different session	Fingers	0.99	0.15	0.85	0.99	0.80
	Wrists	0.97	0.30	0.70	0.98	0.65
	Chest	0.96	0.45	0.55	0.98	0.47

## Data Availability

The data is protected by the GDPR and cannot be publicly available.

## Abbreviations

The following abbreviations are used in this manuscript:

ECG	Electrocardiographic
FCM	Finite-context model
VJ	Vital Jacket
